# Profiling and Bioinformatics Analysis of Differentially Expressed circRNAs in Spinal Ligament Tissues of Patients with Ankylosing Spondylitis

**DOI:** 10.1155/2020/7165893

**Published:** 2020-06-14

**Authors:** Jianqiang Kou, Guoming Liu, Xiangyun Liu, Tianmi Li, Ying Wei, Yuanliang Sun, Ting Wang, Yingzhen Wang, Xiujun Zheng

**Affiliations:** ^1^Department of Orthopedics, The Affiliated Hospital of Qingdao University, Qingdao, Shandong 266000, China; ^2^Department of Operating Room, The Affiliated Hospital of Qingdao University, Qingdao, Shandong 266000, China

## Abstract

Recent studies have reported that circular RNAs (circRNAs) play a crucial regulatory role in a variety of human diseases. However, the roles of circRNAs in ankylosing spondylitis (AS) remain unclear. In this study, we conducted circRNA expression profiling of the spinal ligament tissues of patients with AS by RNA sequencing (RNA-seq) and analyzed the potential functions of differentially expressed circRNA by Gene Ontology (GO) and Kyoto Encyclopedia of Genes and Genomes (KEGG) analyses to investigate the potential mechanisms associated with AS. The results showed that a total of 1,172 circRNAs were detected in the spinal ligament tissue samples, of which 123 circRNAs were significantly differentially expressed by a fold change ≥ 1.5 and *p* value < 0.05. Among these, 57 circRNAs were upregulated, and 66 were downregulated. GO and KEGG analyses demonstrated that the differentially expressed circRNAs were mainly involved in the regulation of biological processes of peptidyl-serine phosphorylation and human immune system that may be related to AS. In addition, the circRNA/miRNA interaction networks were established to predict the potential roles of differentially expressed circRNAs by bioinformatics analysis. Taken together, these results revealed the expression profiles of circRNAs and the potential functions of the differentially expressed circRNAs in the spinal ligament tissue of patients with AS, which may provide new clues for understanding the mechanisms associated with AS, and proceed to identify novel potential molecular targets for the diagnoses and treatment of AS.

## 1. Introduction

Ankylosing spondylitis (AS), which most commonly affects the sacroiliac joint and the axial joint of the spine, is an autoimmune disorder with a global incidence of about 2%-5%, and the patients with AS are often young and middle-aged male [1]. The typical clinicopathological features of AS are inflammation and new bone formation in sacroiliac joint, spine, and peripheral joints (especially hip joint), which finally result in ankylosis [2]. A lot of results have indicated that the inflammation in AS initially occurs at the tendon-bone interface, leading to bone proliferation [2–4]. Progress in the early diagnosis and treatment of AS has been achieved, while the effect of clinical treatment is not as well as people expected. However, many studies suggested that AS has a high heritability [5]; the pathogenesis of AS has been more likely multifactorial and poorly understood to date. Therefore, to elucidate the pathogenesis of AS would be of great value in theory and clinical applications.

Circular RNAs (circRNAs) are a novel class of endogenous noncoding RNAs with a covalently closed circular structure [6–9]. Unlike the linear RNAs, circRNAs have no free 3′-end polyA tail and 5′-end cap, which prevents them from being digested by nucleic acid exonuclease [10]. Therefore, the closed circular structure of circRNAs makes them incredibly stable, and that these may be potentially utilized as molecular markers. In addition, circRNAs possess tissue specificity and are often highly conserved among many species [11]. Recent studies have indicated that circRNAs have diverse biological functions and play crucial regulatory roles in many human diseases [12, 13]. It has been confirmed that circRNAs regulate the expression of miRNA target genes by acting as microRNA (miRNA) sponges [8, 9]. Extensive studies have revealed that numerous miRNAs may be associated with AS, such as miR-29a, miR-335-5p, miR-27a, let-7i, miR-146a, miR-29a, and miR-155 [14, 15]. Besides, a previous study indicated that the aberrant expression of a variety of lncRNAs has also been observed in the peripheral blood of patients with AS [16]. However, there is no report on circRNAs in patients with AS.

Therefore, in this study, we recruited three AS patients as the experimental group and three patients with lumbar disc herniation as the control group and collected their spinal ligament tissues. We performed RNA sequencing (RNA-seq), and then, the differentially expressed circRNAs in the spinal ligament tissue between the two groups were analyzed by bioinformatics analysis. Gene Ontology (GO) and Kyoto Encyclopedia of Genes and Genomes (KEGG) pathway analyses were used to predict the biological functions of the key differentially expressed circRNAs. In addition, the interaction between circRNAs and miRNAs and the networks of circRNA-miRNA was, respectively, predicted and constructed by bioinformatics analysis. This study conducted the circRNA expression profiling of spinal ligament tissues of patients with AS and demonstrated the potential functions of differentially expressed circRNAs, which may provide new clues for studying the mechanisms and potential molecular targets for the treatment of AS.

## 2. Materials and Methods

### 2.1. Patients and Specimens

This study was approved by the Ethics Committee of the Affiliated Hospital of Qingdao University and conformed to the Ethical Guidelines of the Declaration of Helsinki. All of the participants signed informed consent forms upon disclosure of the study details. In this study, the experimental and control groups received posterior lumbar or thoracic decompression and fusion surgery at the Department of Spinal Surgery of TThe Affiliated Hospital of Qingdao University from January 2017 to December 2018. Patients with AS were 64, 58, and 76 years old, and all of them were HLA B27^+^. According to kyphosis deformity, the patients with AS have exhibited symptoms of bilateral damage of the sacroiliac joint in the computed tomography results and spinal and sacroiliac joint fusion in the X-ray results, which completely meet the revised New York AS standard [17]. None of the patients with AS were treated with nonsteroidal anti-inflammatory drugs or biological agents and suffered any complication. The participants in control group were patients with lumbar disc herniation, aged 58, 69, and 64 years. All of the participants had no other type of autoimmune diseases, and the characteristics of all of the participants are shown in Table 1. The spinal ligament tissue samples, including supraspinous and interspinous ligaments, were cut and removed from six participants. The tissue samples were snap frozen in liquid nitrogen and stored at -80°C until analysis.

### 2.2. RNA Sequencing (RNA-seq)

We did sampling once from each patient in each group; that is, three biological repeats/RNA-sequencing in each group and RNA sequencing were used for circRNA expression profiling in the spinal ligament tissue samples from three AS patients and three patients with lumbar disc herniation under the same conditions at a time [18, 19]. According to the manufacturer's instructions, total RNA was isolated from the two group samples using a Magen Hipure Total RNA Mini Kit (Magen, Guangzhou, China). Subsequently, the concentration and integrity of the isolated RNA were determined using the Qubit 3.0 Fluorometer (Invitrogen, Carlsbad, CA, USA) and the Agilent 2100 Bioanalyzer (Applied Biosystems, Carlsbad, CA, USA), respectively. RNA-seq libraries were prepared as previously [20]. Briefly, rRNA was removed from total RNA using a KAPA RNA HyperPrep Kit with RiboErase (HMR) for Illumina® (Kapa Biosystems, Inc., Woburn, MA, USA). The RNA samples were fragmented and reversely transcribed to the first-strand cDNA, and then, the directional second strand was synthesized. After cDNA synthesis, a tail and adapter were ligated onto the purified cDNA, and then, the cDNA was amplified. Subsequently, the cDNA library quality and concentration were evaluated using the Agilent 2100 Bioanalyzer (Applied Biosystems, Carlsbad, CA, USA). For sequencing applications, the qPCR-based KAPA Biosystems Library Quantification kit (Kapa Biosystems, Inc.) was used for quantification of the cDNA library. Finally, the RNA-seq libraries were sequenced on the HiSeq X10 system (Illumina, Inc., San Diego, CA, USA), and 150 bp paired-end (PE150) sequencing was performed on all samples.

### 2.3. Identification of circRNAs

Raw reads with adaptors and low-quality tags were removed, and the remaining clean reads were used in the subsequent analyses [21]. First, using Bowtie2 version 2.1.0, the clean paired-end reads were mapped to the latest UCSC transcript set [22], and using RSEM v1.2.15, gene expression levels were estimated [23]. CircRNA expression was normalized using TMM (trimmed mean of *M* values, CPM > 5). For circRNA expression analysis, the reads were firstly mapped to the genome using STAR [24], and then, the circRNA expression was identified and estimated using DCC [25]. The edgeR program (Bioconductor V3.0; Fred Hutchinson Cancer Research Center, Seattle, WA, USA) was used to identify differentially expressed circRNAs [26]. As previously described [27], we controlled the false discovery rate to yield the q value (adjusted *p* value) to obtain the *p* values and determine the threshold of the *p* value. In addition, the spliced reads per billion mapping (SRPBM) value was used to estimate the fold change of circRNA expression in each sample. CircRNAs meeting the condition of *p* < 0.05 and fold change ≥ 1.5 were considered to be differentially expressed [18].

### 2.4. GO and KEGG Pathway Analyses

As previously reported [8], circRNAs are alternatively transcribed from their parental genes, and according to the location information of circRNAs, parental genes that could be regulated by circRNAs were obtained. The potential functions of the parental genes corresponding to the differentially expressed circRNAs were predicted by GO (http://geneontology.org/), and the related pathways were analyzed based on the latest KEGG (https://www.genome.jp/kegg/pathway.html) database. Significant correlations between the parental genes of differentially expressed circRNAs and their potential functions and pathways were determined based on a threshold of *p* < 0.05 and overlap gene count ≥ 2 [28, 29].

### 2.5. Bioinformatics Analysis of circRNA-miRNA Regulatory Networks

Recently, circRNAs were considered miRNA sponges that regulate gene expression [8, 9]. Therefore, we used the miRbase (http://www.mirbase.org) and miRanda (http://www.microrna.org/microrna/home.do) databases to predict potential miRNAs that are associated with the differentially expressed circRNAs in AS [30, 31]. Based on the interactions between the differentially expressed circRNAs and their target miRNAs, circRNA-miRNA interaction networks were constructed and visualized with Cytoscape version 3.7.1.

### 2.6. Statistical Analysis

Statistical analysis was performed using SPSS 19.0 (SPSS Inc., Chicago, IL, USA). Results were expressed as the mean ± standard deviation. Comparisons between the two groups were tested using two-tailed Student's *t* test. Differences were considered statistically significant at *p* < 0.05.

## 3. Results

### 3.1. Identification of Differentially Expressed circRNAs in AS

**Figure 1:**
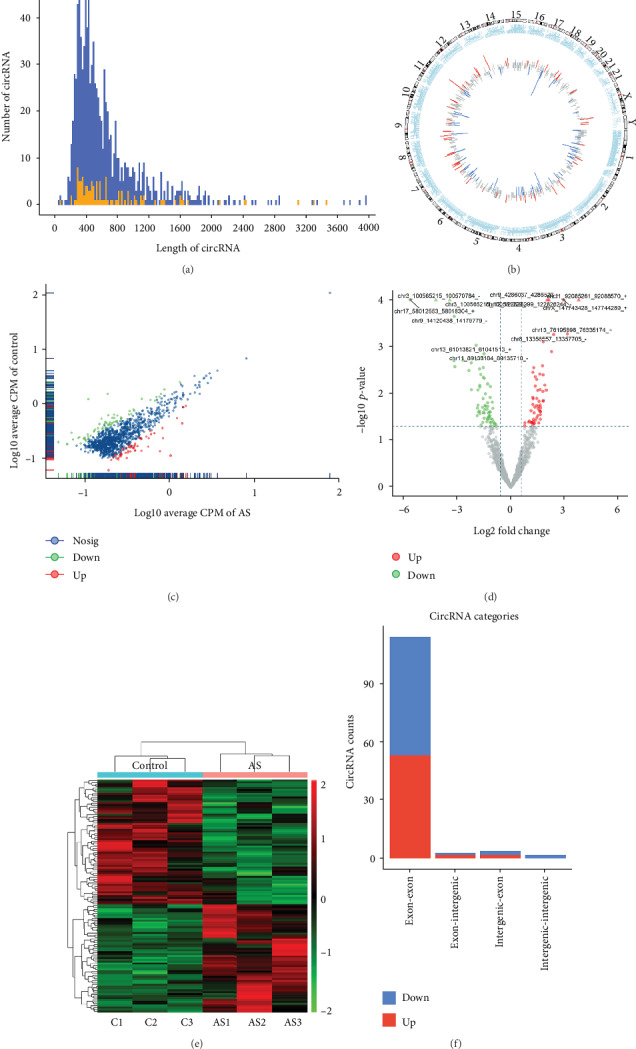
Differential expression profiles of circular RNAs (circRNAs) in spinal ligament tissue from patients with AS and controls by RNA-seq. (a) Length distribution of circRNAs was identified in this study. Orange represents differentially expressed circRNAs with statistical significance (*p* value < 0.05 and fold change ≥ 1.5), and blue represents the circRNAs with no any changes in expression between the two groups (*n* = 3). (b) Distribution of identified circRNAs on human chromosomes. The length of the lines represents the relative size of fold change, and red and blue indicate the upregulated and downregulated circRNAs, respectively. (c) The scatter plot of circRNA expression between the two groups. The dots in the figure represents circRNAs. Compared with the controls, the red dots and green dots, respectively, indicate the upregulated and downregulated circRNA expressions in the spinal ligament tissues of AS patients (*p* value < 0.05 and fold change ≥ 1.5), and the blue dots indicate no change in circRNA expression. (d) The volcano plot depicting circRNA expression profiles in the spinal ligament tissues of the two groups. The two vertical blue lines refer to a 1.5-fold (log2 fold change) upregulation and downregulation, respectively. The horizontal blue line corresponds to a *p* value of 0.05 (−log10 *p* value). Compared with the controls, the red and green dots, respectively, indicate upregulated and downregulated circRNA expressions in the spinal ligament tissues of patients with AS (*p* value < 0.05 and fold change ≥ 1.5). (e) Hierarchical clustering analysis of circRNAs that were significantly differentially expressed (*p* value < 0.05 and fold change ≥ 1.5) in the spinal ligament tissue between the two groups. (f) Categories of differentially expressed circRNAs based on their genomic origin.

To identify differentially expressed circRNAs in patients with AS, RNA sequencing was used for circRNA expression profiling in the spinal ligament tissue samples from three AS patients and three patients with lumbar disc herniation. The main characteristics of all of the patients were presented in Table 1. Sequencing identified a total of 1,172 circRNAs, and the details on total circRNA expression profiling were shown in supplementary data (available here). Approximately 98% of the identified circRNAs were <2,000 nucleotides (nt) in length, and the length of most differentially expressed circRNAs with statistical significance between the two groups was also <2,000 nt (Figure 1(a)). The distribution of identified circRNAs on the human chromosomes is shown in Figure 1(b). Briefly, no differentially expressed circRNAs were observed on chr15 and chr20, and only the upregulated circRNAs from chr12, chr18, chr21, and chrY were transcribed, whereas the upregulated and downregulated circRNAs were transcribed from all other chromosomes except for chr 22, which only had one downregulated circRNA (Figure 1(b)). The scatter plot in Figure 1(c) depicts the variations in circRNA expression levels between the two groups. As shown in the volcano plot, the circRNAs with fold changes ≥ 1.5 and *p* value < 0.05 were considered to be significant differentially expressed between the two groups, and among these differentially expressed circRNAs, 57 circRNAs were upregulated and 66 circRNAs were downregulated (Figure 1(d)). The details of the top 10 circRNAs showing the significant upregulation or downregulation are shown in Table 2. Besides, hierarchical clustering revealed that circRNA expression levels were distinguishable in Figure 1(e). In addition, the vast majority of differentially expressed circRNAs in AS are located in the exonic region (Figure 1(f)). Based on the sequence structure origin of the circRNAs, 114 of the differentially expressed circRNAs in AS were exon-exon circRNAs, 3 were exon-intergenic circRNAs, 4 were intergenic-exon circRNAs, and 2 were intergenic-intergenic circRNAs. In summary, RNA-seq analysis suggests that the expression levels of some circRNAs in the spinal ligament tissue samples had significantly changed between patients with AS and the controls.

### 3.2. GO and KEGG Pathway Analyses of the Differentially Expressed circRNAs in AS

**Figure 2:**
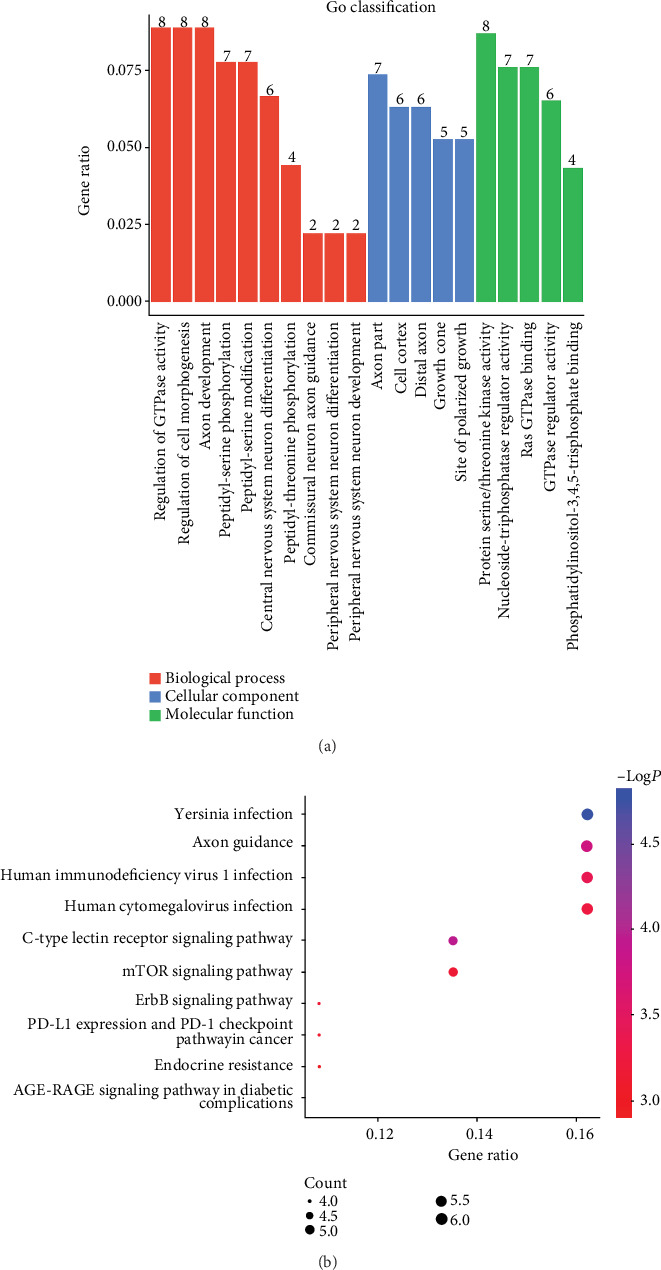
GO and KEGG pathway analyses of differentially expressed circRNA. (a) GO analysis of differentially expressed circRNAs (*p* value < 0.05 and overlap gene count ≥ 2). The horizontal axis is the enrichment score for each GO term, and the vertical axis is the GO term. The enrichment score was calculated as -log10 (*p* value). The number on the histogram columns represents the number of overlapping genes. (b) The KEGG enrichment scatter plot of differentially expressed circRNAs with the 10 highest enrichment scores (*p* value < 0.05 and overlap gene count ≥ 2). The horizontal axis is the enrichment score for each KEGG term, and the vertical axis is the KEGG pathway names.

To explore the functions of differentially expressed circRNAs based on their parental genes in AS, GO and KEGG pathway analyses were conducted to predict the possible functions of circRNAs (*p* < 0.05). GO analysis comprised three major domains: “biological process” (BP), “cell component” (CC), and “molecular function” (MF). The top five enriched GO terms of differentially expressed circRNAs in each domain were “regulation of GTPase activity”, “regulation of cell morphogenesis”, “axon development”, “peptidyl-serine phosphorylation” and “peptidyl-serine modification” in BP; “axon part”, “cell cortex”, “distal axon”, “growth cone” and “site of polarized growth” in CC; “protein serine/threonine kinase activity”, “nucleoside-triphosphatase regulator activity”, “Ras GTPase binding”, “GTPase regulator activity”, and “phosphatidylinositol-3,4,5-trisphosphate binding” in MF (Figure 2(a)). In addition, the detailed information of the enriched GO terms of differentially expressed circRNAs was shown in supplementary data.

KEGG pathway analysis revealed that the differentially expressed circRNAs in AS might mainly be involved in “Yersinia infection”, “human immunodeficiency virus 1 infection”, and “Human cytomegalovirus infection” (Figure 2(b)). Forty-nine pathways related to the functions of 123 differentially expressed circRNAs were identified by KEGG analysis (supplementary data).

### 3.3. Construction of circRNA/miRNA Interaction Networks in AS

**Figure 3:**
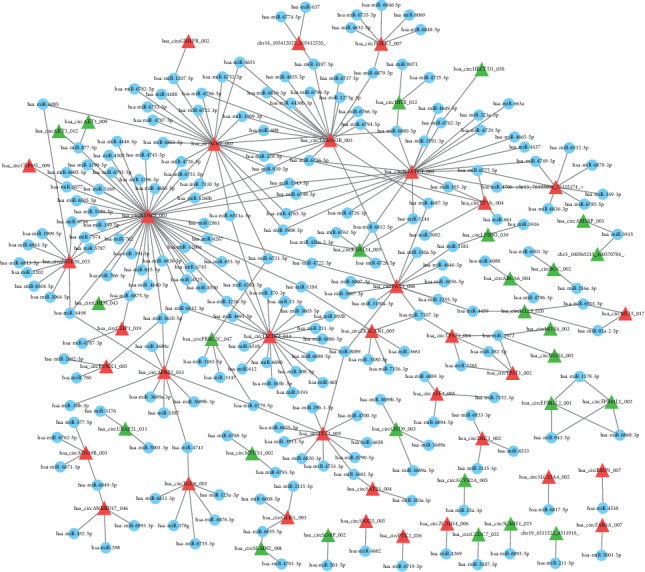
circRNA/miRNA interaction networks for differentially expressed circRNAs. The interaction networks of differentially expressed circRNAs and their complementary binding miRNAs were visualized using Cytoscape 3.7.1 software. The red and green triangles represent upregulated and downregulated circRNAs, respectively, and the blue spots indicate microRNAs. The networks included 60 differentially expressed circRNAs and 221 miRNAs.

Growing evidence shows that circRNAs act as miRNA sponges via miRNA response elements (MREs) that regulate the functions of their target mRNAs. To further evaluate the potential functions of the differentially expressed circRNAs in AS, the circRNA-miRNA networks were predicted using the miRBase and miRanda databases and visualized with Cytoscape version 3.7.1. Studies suggest that the more MREs of one miRNA on one circRNA sequence, the microRNAs are more likely regulated by the circRNAs [8, 9]. In addition, there are many different MREs on one circRNA sequence, and the MRE of one miRNA also exists in a variety of circRNAs' sequence. Given that the networks consisting of the top 300 circRNA-miRNAs were constructed in previous studies [32, 33], the top 300 networks of circRNA/miRNA were selected in this study, and we found that 60 of 123 significantly differentially expressed circRNAs were predicted to interact with 221 miRNAs. The circRNA/miRNA networks were established to clarify the interactions between circRNAs and their target miRNAs (Figure 3). The networks show that hsa_circMNT_002 had five MREs of hsa-miR-6722-3p, and hsa-miR-1972, hsa-miR-4706, hsa-miR-6756-5p, and hsa-miR-6812-5p were found to be regulated by four circRNAs, which is higher than that of other miRNAs. Furthermore, hsa_circRUSC2002, hsa_circMNT002, and hsa_circNFATC1001 were predicted to have complementary binding sites for 45, 24, and 22 miRNAs, respectively, which suggest that the differentially expressed circRNAs play important roles in AS.

## 4. Discussion

In this study, we identified numerous circRNAs that are significant differentially expressed in the spinal ligament tissues of AS patients. RNA-seq analysis showed that 123 circRNAs were differentially expressed in the spinal ligament tissues from AS patients compared with the controls. Among these, 57 circRNAs were upregulated and 66 were downregulated. In addition, we performed GO and KEGG pathway analyses and established circRNA/miRNA interaction networks to predict the potential functions of differentially expressed circRNAs in AS.

AS is a chronic autoimmune disease that is characterized by inflammation and pathological osteogenesis and is associated with HLB-27 and T cells [34, 35]. Although many studies have significantly improved our understanding of the pathophysiology of AS, its etiology and pathogenesis remain unclear. Recent studies have indicated that a numerous molecular and biochemical changes are involved in the cellular mechanisms of AS [3], and increasing evidence suggests that ncRNAs, such as lncRNAs and miRNAs, play important roles in the pathogenesis of AS. Some studies have shown that significant changes in the expression of lncRNAs and miRNAs in AS, and the dysfunction of certain lncRNAs and miRNAs has been associated with the pathophysiology of AS. For example, it has been shown that four lncRNAs, namely, lnc-ZNF354A-1, lnc-LIN54-1, lnc-FRG2C-3, and lnc-USP50-2 are involved in osteogenic differentiation of mesenchymal stem cells (MSCS) in patients with AS [36]. A recent study has revealed that lncRNA-AK001085 is downregulated in patients with AS and considered to be a potential diagnostic molecular marker [37]. In addition, the expression of miR-29a, miR-335-5p, miR-27a, and let-7i is upregulated in peripheral blood monocytes of patients with AS [14], and serum miR-146a, miR-29a, and miR-155 levels in AS patients have also significantly increased [15]. In recent years, more studies have focused on circRNAs. However, to date, information on the function of circRNAs in the spinal ligament tissues of patients with AS is limited. To the best of our understanding of the regulatory mechanism of circRNAs in AS, this is the first study that has investigated changes in circRNA expression profiles in the spinal ligament tissues of patients with AS by RNA-seq and bioinformatics analyses, which could be an important step in elucidating the underlying molecular mechanisms associated with AS.

circRNAs are highly capable of resisting damage caused by RNase and, thus, make them more stable than linear RNAs. Moreover, circRNAs have highly conserved tissue and cell specificity [11]. Recent studies have shown that circRNAs, such as circular RNA Atp9b [38] and circRNA hsa_circ_0005105 [39], are involved in osteoarthritis [40]. Ligaments connect the ends of bones and allow stability and mobility of most joints. Previous studies have revealed that ligaments may be the main target tissue for inflammation and ossification in AS and are responsible for many symptoms of AS patients [17, 41, 42]. Based on the results of this study, we hypothesize that the differential expression of circRNAs in the ligament tissues of AS patients is more specific and sensitive than those in peripheral blood, and thus, we collected spinal ligament tissues of AS patients for circRNA profiling by RNA-seq. We detected a total of 1,172 circRNAs that are located in 46 chromosomes in the spinal ligament tissue samples, and approximately 98% of the identified circRNAs were <2,000 nt in length (Figure 1(a)). Among these differentially expressed circRNAs, 57 circRNAs were upregulated and 66 circRNAs were downregulated (*p* value < 0.05 and fold change ≥ 1.5; Figure 1(d)), and the length of most differentially expressed circRNAs was also <2,000 nt (Figure 1(a)). Recent studies have shown that circRNAs are alternatively transcribed from exons, introns, or other regions of their parental genes [43]. The results of sequencing have shown that the vast majority of differentially expressed circRNAs in AS are located in exonic region (Figure 1(f)). Previous studies have also demonstrated that various types of circRNAs have different functions [44]. The canonical pathway of circRNA biologic regulation involves acting as sponges of miRNAs [8, 9]. Some circRNAs can affect protein function by directly binding to them [6, 45, 46] and translated into proteins [47, 48].

Because circRNAs have been associated with various human diseases [44], these are considered potential clinical diagnostic and therapeutic molecules. To further investigate the circRNA functions of differentially expressed circRNAs in AS, GO and KEGG analyses were performed to predict their possible biological functions and underlying mechanisms. The results of GO analyses have shown that the differentially expressed circRNAs in AS are enriched in BP and MF, such as “peptidyl-serine phosphorylation”, “protein serine/threonine kinase activity”, and “peptidyl-threonine phosphorylation” (Figure 2(a)), which may be involved in the physiological functions of mitogen-activated protein kinase (MAPK) signaling pathways and phosphatidylinositol 3-pinases (PI3K)/Akt signaling pathways that are associated with inflammation-induced apoptosis in chondrocytes, such as IL-1*β* [49]. We found that “regulation of GTPase activity”, “GTPase regulator activity”, and “Ras GTPase binding” are also enriched in BP and MF (Figure 2(a)), while there are few studies about the relationship between GTPase and AS. However, previous studies have shown that GTPase are associated with human diseases [50], which suggested that GTPase may be involved in AS. Moreover, KEGG analysis demonstrated that differentially expressed circRNAs might be related to “Yersinia infection”, “Human immunodeficiency virus 1 infection”, and “Human cytomegalovirus infection”, which are associated with the immune system (Figure 2(b)). In addition, “T cell receptor signaling pathway” and “Th17 cell differentiation” was 11th and 12th in the results of KEGG analysis, respectively (supplementary data). However, previous studies have shown that AS is an autoimmune disorder that is associated with Th17 cells and immune pathways, including the interleukin- (IL-) 17/IL-23 pathway [51, 52], and Th17 cells derived from CD4^+^T cells can release various kinds of cytokines, such as IL-17A and IL-22, which result in bone erosion/proliferation in AS [3], whereas the differentiation of Th17 cells is influenced by multiple inflammatory cytokines, including IL-1*β*, IL-6, TGF-*β*, and IL-23 [52].

Current studies have confirmed that circRNAs in the cytoplasm may play crucial roles in posttranscriptional gene regulation by sponging miRNAs as competing endogenous RNAs or RNA-binding proteins [6, 8, 9, 46]. For example, ciRS-7 has been reported as a miR-7 sponge that is involved in osteosarcoma [53] and affected brain function [54]. The results of the present identified numerous differentially expressed circRNAs in the cytoplasm (Figure 2(a)), and the circRNA/miRNA interaction network analysis was established to further demonstrate the potential functions of the differentially expressed circRNAs. We have also predicted the interactions of the differentially expressed circRNAs in AS with miRNAs by bioinformatics analysis. The top 300 networks of circRNA-miRNA were selected, and we predicted that 60 of 123 significantly differentially expressed circRNA interaction with 221 miRNAs (Figure 3). The networks show that hsa_circMNT_002 has five MREs of hsa-miR-6722-3p, and hsa-miR-1972, hsa-miR-4706, hsa-miR-6756-5p, and hsa-miR-6812-5p are regulated by four circRNAs, which is higher than that of other miRNAs. Furthermore, hsa_circRUSC2002, hsa_circMNT002, and hsa_circNFATC1001 were predicted to have complementary binding sites for 45, 24, and 22 miRNAs, respectively (Figure 3), which suggests that these circRNAs might be associated with the pathogenesis of AS by regulating miRNAs.

## 5. Conclusion

This study has provided the first evidence of circRNA expression profiles in the spinal ligament tissue of patients with AS using RNA-seq, and the results indicated that numerous differentially expressed circRNAs may be associated with AS. Furthermore, the potential functions of these circRNAs were investigated by bioinformatics analysis and circRNA/miRNA interaction networks were constructed. These findings may provide novel clues for understanding the mechanisms and have identified novel potential molecular targets for the diagnoses and treatment of AS. However, to better understand the role of circRNAs in AS, additional investigations are warranted.

## Figures and Tables

**Table 1:** Personal characteristics of participants in the sequencing.

Items	Control group			Experimental group		
	C1	C2	C3	AS1	AS2	AS3
Age (years)	58	69	64	64	58	76
Sex	Female	Male	Male	Male	Male	Male
Clinical symptoms	Lumbar disc herniation	Lumbar disc herniation	Lumbar disc herniation	AS for 40 years, lumbar kyphosis deformity	AS for 8 years, thoracic fracture	AS for 30 years, thoracic fracture and kyphosis deformity
Procedures	Posterior lumbar decompression and fusion	Posterior lumbar decompression and fusion	Posterior lumbar decompression and fusion	Posterior lumbar decompression and fusion	Posterior thoracic decompression and fusion	Posterior thoracic decompression and fusion
HLA B27^+^	No	No	No	Yes	Yes	Yes
Bilateral damage of the sacroiliac joint	No	No	No	Yes	Yes	Yes
Spinal and sacroiliac joint fusion	No	No	No	Yes	Yes	Yes
Non-steroidal anti-inflammatory drugs	No	No	No	No	No	No
Other type of autoimmune disease.	No	No	No	No	No	No
Complications	No	No	No	No	No	No

**Table 2:** The top 10 circRNAs with the significant upregulation or downregulation.

Position	circBankID	circBaseID	Gene symbol	Length	Type	log2FC	*p* value	*p*_Adj_	Regulation
chr11_92085261_92088570_+	hsa_circFAT3_006	hsa_circ_0000348	FAT3	3309	Exon-exon	3.7819	0.0000	0.0058	Up
chr13_76195898_76335174_+	NA	NA	LMO7	NA	Exon-exon	3.1527	0.0005	0.0639	Up
chrX_147743428_147744289_+	hsa_circAFF2_005	hsa_circ_0001947	AFF2	861	Exon-exon	2.9490	0.0000	0.0000	Up
chr8_13356557_13357705_-	hsa_circDLC1_002	hsa_circ_0135780	DLC1	1148	Exon-exon	2.3865	0.0005	0.0639	Up
chr2_200233327_200298237_-	hsa_circSATB2_015	hsa_circ_0003915	SATB2	531	Exon-exon	2.2778	0.0013	0.1144	Up
chr9_4286037_4286523_-	hsa_circGLIS3_003	hsa_circ_0002874	GLIS3	486	Exon-exon	2.1186	0.0000	0.0047	Up
chr12_122825299_122826244_-	hsa_circCLIP1_019	hsa_circ_0029069	CLIP1	945	Exon-exon	2.0273	0.0001	0.0123	Up
chr3_18419661_18462483_-	hsa_circSATB1_004	hsa_circ_0064555	SATB1	1599	Exon-exon	1.9818	0.0084	0.2278	Up
chr21_36206706_36231875_-	hsa_circRUNX1_005	hsa_circ_0002360	RUNX1	297	Exon-exon	1.8979	0.0066	0.2154	Up
chr9_37424841_37426651_+	hsa_circGRHPR_002	hsa_circ_0001861	GRHPR	321	Exon-exon	1.8159	0.0008	0.0828	Up
chr6_144858717_144864006_+	hsa_circUTRN_065	hsa_circ_0130908	UTRN	362	Exon-exon	-2.1886	0.0082	0.2278	Down
chr19_4511522_4511918_-	NA	NA	PLIN4	NA	Exon-exon	-2.1979	0.0022	0.1357	Down
chr8_17570722_17581342_-	hsa_circMTUS1_004	hsa_circ_0083443	MTUS1	336	Exon-exon	-2.3338	0.0032	0.1500	Down
chr5_102432244_102433485_-	hsa_circGIN1_005	hsa_circ_0006499	GIN1	655	Exon-exon	-2.6637	0.0019	0.1295	Down
chr1_154207066_154207767_+	hsa_circUBAP2L_011	hsa_circ_0110845	UBAP2L	298	Exon-exon	-3.1140	0.0027	0.1426	Down
chr9_14120438_14179779_-	hsa_circNFIB_012	hsa_circ_0138300	NFIB	683	Exon-exon	-3.1464	0.0002	0.0326	Down
chr10_34558584_34573173_-	hsa_circPARD3_036	hsa_circ_0018168	PARD3	354	Exon-exon	-3.1686	0.0020	0.1295	Down
chr3_100565215_100581229_-	hsa_circABI3BP_001	hsa_circ_0121334	ABI3BP	513	Exon-exon	-3.4042	0.0000	0.0000	Down
chr3_100565215_100570784_-	NA	NA	ABI3BP	NA	Exon-exon	-4.1847	0.0000	0.0000	Down
chr17_58012553_58018304_+	NA	NA	RPS6KB1	NA	Exon-exon	-5.5909	0.0000	0.0079	Down

Position: chromosome location of circRNA; log2FC: log2Fold change; *p*_Adj_: adjusted *p* value; NA: not available.

## Data Availability

The datasets used and/or analyzed during the current study are available from the corresponding author on reasonable request.

## Supplementary Materials

Supplementary Materials Sequencing identified a total of 1,172 circRNAs, and the details on total circRNA expression profiling were shown in supplementary data.
